# Interest in digital health tools for miscarriage support: A qualitative assessment of Canadian women facing early pregnancy loss

**DOI:** 10.1177/17455057241311424

**Published:** 2025-01-25

**Authors:** Breanna Flynn, Anjali Sergeant, Genevieve Tam, Megan Gomes, Roopan Gill

**Affiliations:** 1 Department of Obstetrics and Gynecology, University of Ottawa, Ottawa, ON, Canada; 2 Department of Obstetrics and Gynecology, The Ottawa Hospital, Ottawa, ON, Canada; 3 Department of Medicine, University of British Columbia, Vancouver, BC, Canada; 4 Vitala Global Foundation, Vancouver, BC, Canada; 5 Department of Obstetrics & Gynecology, University of Toronto, Toronto, ON, Canada

**Keywords:** Spontaneous abortion, miscarriage, mobile health, digital health, pregnancy support, user-centered design

## Abstract

**Background:**

Early pregnancy loss (EPL) occurs in 10%–15% of all pregnancies but remains an underrecognized and undertreated condition. In Canada, resources to support individuals and their partners facing EPL remain scarce despite a high burden of psychosocial sequelae. Digital health tools hold the potential to fill important gaps in reproductive healthcare.

**Objectives:**

We sought to better understand the perspectives of individuals who experienced pregnancy loss and explore how digital health tools could offer support.

**Design:**

We conducted a qualitative study with grounded theory methodology to address our objectives.

**Methods:**

The study was conducted between September 2021 and April 2022 in Ottawa, Canada. Participants between 18 and 45 years of age who resided in Canada and experienced EPL up to 12 + 6 week gestation within the last 2 years were included. Enrolled participants who provided informed consent completed a single in-depth interview. Data were analyzed iteratively by two trained research team members with thematic techniques supported by NVivo software.

**Results:**

Interviews were conducted with 14 participants who had experienced EPL. All participants identified as female and resided in Canada, with 28.6% (*n* = 4) between 26 and 30 years of age, and the remaining 71.4% (*n* = 10) between 31 and 40. Qualitative analysis identified three primary themes centered around participants’ experiences of miscarriage, access to information and support for EPL in Canada, and desires and preferences for a digital miscarriage tool.

**Conclusion:**

Miscarriage is an emotionally difficult experience for women and their loved ones, who often do not receive timely and compassionate care within the healthcare system. Participants were highly motivated to co-develop a digital intervention for EPL that is designed to fill gaps in care. The digital companion would assist individuals through their miscarriage journey by providing evidence-based and locally relevant medical information as well as avenues to access both professional and informal forms of psychosocial support.

## Introduction

While not uncommon, miscarriage can be a difficult and isolating experience for individuals and their loved ones. Miscarriage occurs in approximately 10%–15% of all early pregnancies, most often due to fetal chromosomal abnormalities.^1–3^ The American College of Obstetricians and Gynecologists defines early pregnancy loss (EPL) as a “nonviable, intrauterine pregnancy” that occurs within the initial 12 weeks, or first trimester, of gestation.^1^

Many people facing miscarriage experience both acute physical symptoms and challenging emotions around loss. An estimated 20% of women experience significant anxiety, depression, and/or post-traumatic stress disorder which may persist for months to years after EPL.^4,5^ At a population level, the long-term mental health sequelae associated with EPL result in decreased functioning and workplace productivity.^3^

Despite the fact that approximately 1 in 10 women is affected by EPL during the life course, the experience is often minimized by healthcare professionals.^3^ In healthcare settings, EPL may be dismissed as normal, unavoidable, or not grief-worthy, contributing to harmful misconceptions around EPL.^6,7^ The dearth of high-quality and comprehensive miscarriage care exacerbates the physical and psychosocial challenges associated with the experience.^8–10^ A 2021 Lancet series on miscarriage highlights the need for a global re-think around healthcare services and attitudes on this public health issue.^11^

In the Canadian setting, miscarriage care is limited by scarce resources and siloed pathways for patients to seek support, with particular gaps in mental health services.^10,12^ Previous research has shown that the majority of Canadians facing pregnancy loss seek care in the emergency department where they do not receive adequate psychosocial support or follow-up.^10^ While technology cannot replace the importance of direct provider support, digital tools may help to bridge gaps in psychosocial EPL care.^13^ Evidence suggests that digital health tools may effectively support sexual and reproductive health needs by providing evidence-based and individualized information to users.^14–17^ In this emerging field, there is little research focused on digital health tools, specifically designed for people experiencing EPL.

Given the immense need for high-quality and patient-centered miscarriage care in Canada and beyond, we sought to better understand the needs and desires of patients who experienced EPL. Specifically, we focused on the ways in which a digital health tool could offer support and information to women experiencing pregnancy loss. To gain an in-depth understanding of patient’s experiences and perspectives, we conducted a qualitative analysis with in-depth interviews focused on our two primary objectives:

1. Outline the experiences, desires, concerns, and needs of people experiencing EPL in Canada.
2. Determine how digital health tools can support individuals facing EPL.

## Methods

### Design

In order to address our study objectives, we conducted a qualitative research study involving in-depth interviews with individuals who experienced EPL in a Canadian setting. The study was conducted between September 2021 and April 2022. This represents phase II of a mixed-methods project focused on pregnancy loss and digital health tools; phase I involved an exploratory quantitative survey with a similar population.

Qualitative research through semi-structured interviews were chosen to offer participants increased autonomy in how they choose to share their stories and to obtain a rich diversity of participant perspectives.^18,19^

We adopted a grounded theory approach to qualitative analysis, which allows researchers to identify pertinent themes and construct theory based on participant data.^20^ The inductive approach of this methodology is well-suited for research questions which have not already been explored comprehensively in the literature and offers flexibility when conducting research with underserved or difficult-to-reach populations.^21^ These strengths pertain to our study population, as those facing EPL may have faced negative healthcare experiences or may feel that their experiences of loss are not significant. The study was designed in accordance with the Consolidated Criteria for Reporting Qualitative Research (COREQ) guidelines, to ensure rigor in research methods and reporting.^22^

### Participants

Qualitative research participants were recruited from the phase I survey study, which included individuals aged 18–45 who self-reported to have experienced EPL up to 12 + 6 week gestation in the preceding 2-year period. The inclusion criteria also required individuals to reside in Canada and understand English. Participants who had a therapeutic abortion or ectopic pregnancy were excluded. For the phase I study, participants were recruited to complete an online survey via social media advertisements (Instagram and Facebook) and hospital/clinic posters. The 185 participants who were included and completed the survey were offered the choice to provide contact information to participate in the phase II qualitative study.

### Data collection

Individuals who wished to participate in phase II were contacted by email and an interview was scheduled via telephone or audio-only teleconference based on participant preference. Interviews were conducted by one of two members of the research team (BF) and (RG). Prior to starting the interview, verbal consent to participate and have the interview audio-recorded and transcribed was obtained by the researchers by reading an ethics board-approved Verbal Consent Script to each participant. The Verbal Consent Script contained information on the research team, goals of the study, potential harms/benefits of participation, and privacy/confidentiality. Both parties confirmed that no one else was present during the interview. Participants completed a single interview session of approximately 60 min in length. A qualitative interview guide was utilized by interviewers, which included topics on the experience of miscarriage and healthcare received, methods of accessing health information, interest in using a digital tool to support care during and after EPL, and preferences for the design of a digital intervention. All participant data were de-identified and stored on an encrypted institutional server at The Ottawa Hospital.

The recruitment target for this study was set flexibly between 10 and 20 participants, with the goal of achieving sufficient information power for qualitative research.^23^ As interviews were conducted, data were reviewed iteratively until saturation of themes was reached.

### Data analysis

The transcripts from participant interviews were stored and analyzed in NVivo software using thematic analysis techniques. Transcripts were read and coded (BF) and (RG), by two female clinician-researchers with training in qualitative interview methods. Inductive analysis was performed in order to identify emerging themes from the data, and a coding tree was utilized to organize themes. Segments of interview transcripts were coded into nodes representing similar or repeating ideas, and nodes representing a similar theme were then grouped to develop a thematic map. Divergent participant perspectives were included in the reporting of the results. Due to the limited study period and funding, participants did not have a chance to review the interview transcripts or analyzed results but were interested in receiving information about any related publications.

### Ethical considerations

The study was approved by the Ottawa Health Science Network Research Ethics Board (Protocol ID: 20210460-01H). Participants volunteered to participate and were not compensated.

## Results

### Qualitative interviews

One hundred and eight participants consented to receive information about the phase II qualitative study. The first 14 participants to respond to a formal email about the qualitative portion of the study were recruited, and all completed the study. All participants identified as female, with 28.6% (*n* = 4) between 26 and 30 years of age and the remaining 71.4% (*n* = 10) between the ages of 31 and 40. The majority of participants were married or in a domestic relationship (*n* = 12, 85.7%). All participants resided in Canada at the time of study, though 28.6% (*n* = 4) of participants were born outside of Canada. The majority of participants (*n* = 11; 78.6%) reported that they had a university-level or professional degree.

**Figure 1.**
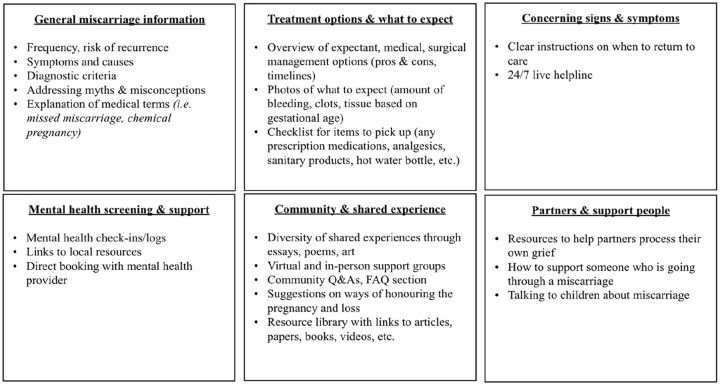
Desired features of a digital health tool.

Three specific themes arose from the qualitative interview analysis: participants’ experience of miscarriage; methods of accessing healthcare information for EPL; and desires and preferences for an evidence-based digital health tool for information about miscarriage. Themes and sub-themes are detailed in Table 1. Participant desires and suggestions for an EPL digital health tool are summarized in Figure 1.

**Table 1.** Qualitative themes, organized by relevant codes and sub-codes.

1. Participants’ experiences of miscarriage a. People experience psychosocial distress and confusion following miscarriage • Misconceptions around miscarriage were commonly encountered ○ Public conceptions of miscarriage exacerbated feelings of self-blame • Mental health difficulties prevalent following miscarriage ○ Participants described anxiety, depression, panic attacks, and sleep disturbances ○ Mental health challenges perpetuated feelings of isolation 2. Access to information and support for EPL in Canada a. Many people face barriers in seeking support within the healthcare system • Frequent dismissal of the miscarriage experience from healthcare workers ○ Participants often did not feel they received high-quality miscarriage care, citing a lack of compassion and understanding from providers • Women did not receive comprehensive information for decision-making ○ Incomplete information around treatment options (i.e. need for evacuation surgery) ○ Self-advocacy was required to receive follow-up or ongoing care b. Digital sources are commonly used to access health information and support • Digital sources offer increased accessibility and immediate information ○ Participants consistently searched for medical information around miscarriage online despite concerns around misinformation • Online sources offered a sense of community and validation 3. Desires and preferences for a digital miscarriage tool a. High degree of interest in an evidence-based digital tool to support miscarriage • Desire for information co-developed with experts in the field ○ Interest in a comprehensive digital health tool that includes information around causes, prevalence, diagnosis, symptoms, and management of EPL • Locally relevant and culturally inclusive information and language within the tool b. Psychosocial support should be embedded within a digital tool • Desire to improve emotional support among individuals experiencing pregnancy loss ○ Resources to encourage reflection and access professional resources ○ Support user interaction within the app and support groups to share experiences around miscarriage • Incorporate information for loved ones on how to best support themselves and the person who is physically miscarrying c. User-friendly design features are highly valued • App or mobile-friendly website design is preferred ○ Desire for simplicity and individualized notification settings

EPL: early pregnancy loss.

### Participants’ experiences of miscarriage

#### People experience psychosocial distress and confusion following miscarriage

Among all participants interviewed, the pregnancy loss was a difficult personal experience that brought on complex emotions. Women consistently reported feelings of self-blame around the miscarriage, and frequently asked themselves what they did to provoke their miscarriage. The guilt and shame they experienced often lasted beyond the physical symptoms:

“I felt so much guilt about the whole process, which I still have and I’m still trying to process.”—Participant ID 13

In addition to commonly expressed feelings of guilt, the miscarriage experience also led many to feel anger and frustration toward their own bodies. One participant illuminated how her psychological distress was tied to personal expectations of what it means to be a woman:

“I kind of felt I was a failure. [Pregnancy] is the one thing that as women we’re supposed to do.”—Participant ID 1

As depicted above, the pressure for women to complete a healthy pregnancy and the sense of failure if they do not is informed by societally mediated gender roles and stereotypes. Publicly held notions of femininity and fertility created an impossible standard for many women who had experienced pregnancy loss.

Furthermore, participants described that many myths and misconceptions about miscarriage continue to permeate our culture. For example, many participants discussed the idea that a pregnant person causes the miscarriage through “irresponsible” activities such as lifting something heavy, exercising or taking a hot bath.

Participants often described feeling isolated or alone after experiencing miscarriage. Due to the fact that many women blamed themselves for the pregnancy loss, guilt, and shame often prevented them from sharing their emotions. As one participant describes:

“I was ashamed that I was going through it, and I didn’t want to tell people how I was feeling.”—Participant ID 11

The isolation was compounded by misconceptions that miscarriage is rare or caused by a personal deficiency. The majority of participants reported that they did not realize that miscarriage is a relatively common experience. Without adequate information, many women continued to blame themselves for the pregnancy loss and experienced difficult emotions without adequate support. For most participants, the initial emotions around loss transformed into longer-standing mental health difficulties associated with the miscarriage experience. Most commonly, women described features of anxiety and depression which for some lasted for years:

“The anxiety was crushing, and it would strike me at the most unexpected times.”—Participant ID 6

“Two years later, [I am] still going through bouts of depression. I’m like, stuck on repeat or something.”—Participant ID 4

As depicted in the above accounts, many participants described that depressive or anxious states would arise unpredictably for prolonged periods following the pregnancy loss. Panic attacks, nightmares, and sleep disturbances were also reported. The feelings of self-blame, guilt, and isolation that all participants shared following miscarriage often preceded longstanding mental health challenges.

### Accessing information and support during and following EPL

#### Many people face barriers in seeking support within the healthcare system

While experiencing distress around their miscarriage, many participants reported difficulties in accessing timely and compassionate healthcare. Women frequently described being dismissed or overlooked by healthcare professionals. As described by participants who sought miscarriage care in the emergency department:

“I was told: Why are you here? Why are you overreacting? It’s just some blood.” — Participant ID 4

“Basically, I just bled in the waiting room, and by the time I saw the doctor I had fully miscarried.”—Participant ID 3

The lack of compassion and understanding from healthcare professionals added to the traumatic nature of the experience. Instead of receiving comprehensive information about the process, participants often described short encounters with healthcare professionals where they felt rushed to make decisions regarding their care. Many highlighted that they lacked knowledge of the different treatment options for miscarriage even after seeking healthcare. As one participant noted, the need for surgery was startling and difficult to comprehend:

“I didn’t realize I would have to have surgery, that was kind of a slap in the face.”—Participant ID 1

Multiple participants reported that the use of medical terminology to describe their miscarriage was difficult to understand, and sometimes invalidating:

“The whole idea of a ‘missed’ miscarriage is something that I wasn’t familiar with until going through it . . . that was kind of a shock for me.” — Participant ID 9

“[The doctor] was referring to the pregnancy as a chemical pregnancy. . . that felt like it was just a made-up pregnancy . . . I was really confused about that term.”—Participant ID 4

Participants agreed that the use of medical terminology without adequate explanation made the experience more distressing. Misperceptions around the term “abortion” used for miscarriage of a wanted pregnancy exacerbated feelings of guilt and self-blame for some participants. For some women, such interactions with health professionals highlighted their own lack of knowledge around the process. Others described that medical terms contributed to the perception that their feelings around loss were unworthy or overly dramatic. Unfortunately, many participants described that the lack of compassion and patience from healthcare professionals during the acute miscarriage period worsened their distress.

Most participants agreed that an acknowledgement of the loss by a healthcare professional would have greatly improved their healthcare experiences during miscarriage. A few participants did have positive experiences seeking care and were meaningfully impacted by providers who displayed empathy and spent time talking about the miscarriage process:

“The people that were amazing were the ones that just took a little bit more time to talk to me as a person instead of just someone coming through the system.”—Participant ID 13

As illuminated in the above quotation, the simple recognition of a person’s humanity can offer solace during this difficult experience. Similarly, those who were able to access mental health support reported great benefits:

“I searched high and low, it took me a good week of research to find a therapist, but it’s finding one that specializes in [pregnancy loss] and that’s exactly what I was looking for. . .it’s helped tremendously.”—Participant ID 11

Participant accounts indicated that psychosocial support is greatly needed in the period following miscarriage, and this support can be extremely valuable. Large gaps exist in the accessibility of mental healthcare in Canada after miscarriage completion and access to care may be particularly difficult for those who cannot afford to pay for private services. Women consistently expressed a desire to be listened to after a particularly distressing event in their lives.

#### Digital sources are commonly used to access miscarriage information and support

When asked about where they accessed information about pregnancy loss, all participants reported using digital technologies, particularly internet search engines. In a context where participants described barriers to timely and comprehensive medical care, digital sources offered easily accessible and immediate information. As one participant described:

“Definitely Dr. Google here is the first step.”—Participant ID 8

While all participants eventually sought medical care, many turned to the internet as the initial means of identifying the signs and symptoms of pregnancy loss. Women who utilized digital websites for medical information highlighted that not all sources are equal; several exercised caution when interpreting the information they accessed online:

“I mean . . . the really heavy bleeding was starting, I think I probably just Googled, which I can see the pros and cons of and . . . I try to use authoritative sites.”—Participant ID 5

As expressed in the quote above, participants were aware of potential medical misinformation online and sought out websites that appeared more professional and evidence-based. However, many still expressed concern that this information may not be entirely accurate or applicable to their specific situation. Interaction with online sources focused on pregnancy loss did not replace the value of speaking with a medical professional.

In addition to seeking out medical information online, some participants also noted that digital sites allowed them to read about miscarriage experiences and stories of others. The period following pregnancy loss was often characterized by loneliness, and these sites offered participants a sense of community:

“I think stories are so powerful to us, like reading someone’s story can make you feel so much less alone.”—Participant ID 4

By interacting with the stories of others through digital media, some participants described that they felt more validated in their own experiences. Participants learned that they were not alone. For some women, this provided a sense of hope and support during a particularly challenging experience that was not available within their traditional social circles.

### Desires and preferences for a digital miscarriage tool

#### High degree of interest in an evidence-based digital tool to support miscarriage

All participants expressed interest in a digital tool designed to support care following pregnancy loss. After experiencing difficulty accessing reliable information and compassionate care during their own miscarriages, many reported that they would have wanted additional resources to assist with decision-making and informing care. As one participant described:

“Having [a tool] at my fingertips, having the resources would have been super helpful at the beginning. And I know that I personally would have benefitted.”—Participant ID 11

Participants were interested in a digital tool that they could trust; there was a high value placed on evidence-based and reliable information about EPL that was sometimes challenging to access online or within healthcare settings. Many noted that the potential benefits of a digital tool would not replace direct care from a healthcare professional but instead could be used as an adjunct:

“It doesn’t replace the time you can spend with your doctor and have things explained to you . . . that it would be in addition to that, and it wouldn’t take away time from it.”—Participant ID 7

In fact, rather than envisioning the tool as separate from the medical care they received, many vocalized that a tool co-created by physicians would increase its appeal. One participant explained that digital information approved by physicians would have been useful during her miscarriage:

“I think it could have been helpful to have information available that I know is legitimate because it’s been created and written by actual doctors. . . not just random information on the internet.”—Participant ID 3

Other participants echoed that they would appreciate a tool that was developed with physicians, specifically medical professionals who practice in Canada and hold expertise in women’s health. In order to ensure that the information provided is locally relevant and discusses resources that are available to Canadians, one participant suggested:

“I would want to make sure that it’s local, like Canadian information, because every country is so different and the information they provide is so different.” –Participant ID 11

The importance of centering local context within a digital tool was a common theme. Amidst a diverse Canadian population, there are a multitude of languages and cultures that participants felt should be considered in miscarriage resources:

“People who don’t really speak English, I feel like they need more support in terms of having proper information . . . I think having the official resource in a number of languages [and] conscious of a number of cultures.”—Participant ID 12

When discussing how a digital tool for EPL information should be developed, participants highlighted the importance of evidence-based and culturally relevant information. The participant interviews emphasized the need for adaptability—that information and resources should be tailored to the local environment and translated into several languages. Implicitly, by offering their own unique opinions and perspectives, participants highlighted the need for co-development with individuals who had experienced EPL.

Participants desired that a digital tool include content on the prevalence, causes, diagnostic criteria, and typical signs and symptoms of EPL. Some suggested that a section explaining commonly used medical terminology around miscarriage would increase their confidence in engaging with medical care. Several participants also thought that it would be useful to have common myths and misconceptions around pregnancy loss debunked. Much of the content that participants desired in a digital miscarriage tool was summarized by one woman who reflected on her experience:

“I think I would have appreciated some facts and information and . . . an explanation as to why or how it would happen, or what the likelihood of it was of happening again, or what my body would feel like going through it.”—Participant ID 10

As alluded to above, participants often described a desire for content that would help them prepare for what to expect during the miscarriage process. This included more information on the physical aspects of the experience:

“I was surprised at how painful [the miscarriage] was . . . that was definitely not what I expected.”—Participant ID 8

This participant was not alone in feeling unprepared for the pain that she experienced during her miscarriage. By including information about common symptoms and how best to manage them, participants thought that a digital tool would have better supported them during the acute phase of the pregnancy loss:

“What does normal bleeding and discharges look like five days after a miscarriage? When should it be concerning?”—Participant ID 2

Miscarriages are often emotionally charged, and it can be challenging for individuals to decipher normal from abnormal symptoms. Participants wanted clear instructions on what signs or symptoms would be concerning and warrant a return to medical care. Relatedly, individuals also wanted more information about miscarriage management options including an outline of the main treatment options available, and the benefits and drawbacks of each. It was also suggested that having a checklist for items to pick up when self-managing the miscarriage, such as analgesics and sanitary products, would be beneficial.

#### Psychosocial support should be embedded within a digital tool

Participants consistently reported that psychosocial distress during and after EPL was the most difficult aspect of the experience. Many desired additional support and resources to seek mental healthcare, which they felt could be supported through a digital miscarriage tool. In terms of specific features, participants unanimously liked the idea of having daily mental health check-ins where the app would prompt you to reflect and record how you are feeling. Direct booking within the app with a counsellor/therapist that is trained specifically for pregnancy loss was another highly desired feature. Additionally, participants suggested that the tool include a list of local therapists based on the user’s location.

Individuals who had experienced pregnancy loss often greatly valued interpersonal support with others with similar experiences. Participant interviews often emphasized the psychosocial benefits of sharing experiences and stories with others:

“It’s hard opening up like that, the more you do, the more you realize there’s other women and men suffering as well.”—Participant ID 14

“Having that resource to connect with people that are going through the same thing is huge for me.”—Participant ID 11

Many participants suggested that a digital miscarriage tool could help people form support networks and build community during this difficult experience. Various ideas were brought forth. Some women suggested that the tool could offer virtual support groups and/or offer links and information surrounding local in-person support groups. Others suggested that the tool included a section dedicated to sharing stories of pregnancy loss through writing and art:

“I love [the] idea because if you know someone wrote a poem that helped them go through their miscarriage, then maybe it would help someone else reading that poem.”—Participant ID 13

The ability to connect with others through a digital tool was a highly desired potential feature that addressed many participants’ experiences of isolation following miscarriage. Engaging with the stories of others offers the potential to offer solace and hope for the future. However, a few participants were concerned that certain narratives around miscarriage may be difficult to hear, especially for those who have experienced recurrent pregnancy loss:

“I’ve read some of those [stories] and. . . it’s helpful to some extent, but it also breaks my heart even more because there are more stories with happy pregnancies after and happy kids after. And it just brings me down even more because I don’t have it . . . it makes me feel even more alone. So, I would be cautious about that.”—Participant ID 7

This participant highlighted the importance of fostering inclusive digital stories, that contain diverse narratives around miscarriage. In addition to the connectedness that could be fostered with others engaging with a digital miscarriage tool, users could share useful information, creative works, and support resources.

In addition to desiring support for their own psychosocial needs, several participants desired more resources for their partners and families during the miscarriage process. Some participants highlighted the difficulties that their partners faced throughout the pregnancy loss process:

“I went through it physically, but it was just as emotionally hard for my husband too, because he lost his child too. . . there should be something there to help spouses.”—Participant ID 13

As depicted in the above quote, a digital tool could offer important support and advice for the loved ones of the individual who is miscarrying. In a context where very few resources for support persons exist, a digital support tool could recognize the difficulty that many loved ones face and offer important information and resources for support. Given that each individual experiencing pregnancy loss has a unique support system that may or may not involve a romantic partner, one participant highlighted the importance of inclusivity around the term “partnership”:

“[I want] a section [of the tool] for partners. . . whether it’s a friend, or romantic partner . . . I know that my husband felt completely and utterly lost and helpless and didn’t really understand the emotions that I was experiencing, but also didn’t know how to support me in the physical aspects, so I think that would be huge.”—Participant ID 6

Many noted that the people in their support networks did not always know how to offer helpful kinds of support. For instance, many wanted their loved ones to avoid making well-intentioned but invalidating comments such as: “at least it was early” or “this happens to everyone.” A digital tool could help loved ones process their feelings around pregnancy loss and also better care for the individual who is physically miscarrying.

#### User-friendly design features are highly valued

Participants consistently expressed that a digital tool would have greater uptake among those miscarrying if it employed a user-friendly design. Most desired that the tool should be accessible on a smartphone and were particularly interested in a mobile app. The majority of patients desired that the tool provides medical information and access to psychosocial support seamlessly:

“Simplicity is the key. Not having too many links to click.”—Participant ID 1

In addition to the simplicity in its design, participants agreed that notifications from the tool should be optional and customizable. Participants discussed that users should be able to control the frequency of notifications based on their individual preferences and their timeline of recovery following the miscarriage:

“I would like to see something with options. I can opt into notifications or out of the notifications.”– Participant ID 2

“. . .Sometimes you don’t want to be distracted by it again, let’s say you’re feeling good that day and then you get a prompt that reminds you [of the loss].”—Participant ID 4

Several participants expressed their desire for customizable notifications, partially because notifications might be unhelpful reminders of the miscarriage or pop up at inconvenient times. For others, notifications from the digital tool were envisioned as useful prompts to check in on an individual’s physical and emotional recovery from miscarriage. As one participant explained:

“I think having something that [asks]: ‘Hey, checking in, how are you now?’ ‘How are you feeling?’ . . . ‘Has your period returned?’”—Participant ID 14

By reminding users to reflect on their symptoms and concerns, a digital tool might allow individuals to keep in touch with themselves throughout the process and recognize when to ask for help. In order to receive individualized and real-time support, some participants desired that the digital miscarriage tool include a 24/7 helpline to connect with a trained professional to provide reassurance or guidance based on their symptoms.

Participants were asked if they would have any security concerns when using/downloading an app dedicated to miscarriage. The vast majority had no concerns at all related to digital security and privacy. A few participants did note the importance of protecting any personal or health information disclosed in the app. While privacy and security were not major themes that arose from participant interviews, the need to maintain a high standard of privacy around health information was made apparent in these few divergent participant views.

## Discussion

Our findings highlight the potential for a digital health tool to address critical gaps in care for individuals experiencing EPL. By better understanding the needs and desires of women facing EPL in Canada, we have created a framework for such a digital support tool.

Women in our study frequently turned to digital health resources for medical information and emotional support, which aligns with high rates of digital engagement reported in this population.^24–26^ Digital tools can play a complementary role in healthcare by bridging gaps in conventional services, offering timely and accessible information, and facilitating more informed interactions between patients and providers.^27,28^

Study participants were interested in a digital tool that provides comprehensive and evidence-based information for miscarriage, reflecting findings in other studies that women value healthcare expert approval of pregnancy loss resources.^27,29^ Women also desired a digital resource that fosters a sense of community with others who have experienced similar losses. Existing evidence highlights the value of informal social support found through online forums and social media in helping individuals process difficult emotions and feel less isolated after miscarriage.^30,31^ A co-designed digital intervention could uniquely provide both interpersonal support from peers and evidence-based medical information from providers, thereby complementing traditional healthcare and addressing the gaps in psychosocial care.

Furthermore, by covering all aspects of the miscarriage experience in one resource, women envisioned a streamlined, trustworthy alternative to navigating overwhelming online content—a challenge documented in studies on digital health information overload.^32^

Accessibility and inclusivity were recurring themes, with participants advocating for a resource offering clear, culturally sensitive, and locally relevant information. This aligns with existing evidence that healthcare resources must consider the social determinants of health and involve end-users in the development process to ensure equity and effectiveness.^33,34^

The development of a digital miscarriage resource presents an opportunity to fill a significant void in EPL care. By providing 24/7 access to evidence-based information, mental health resources, and community support, a customizable digital intervention has the potential to improve the care of women facing EPL at a population level.

### Limitations

The study was limited by sampling bias, as participants who were recruited took part in a survey that was primarily advertised in urban healthcare centers that may not be accessible to women in rural or non-biomedical care settings. Women were more highly educated in comparison to the general Canadian population. Previous studies have demonstrated that highly educated women are more likely to accurately identify facts about miscarriage.^35,36^ This population may also be more inclined to use digital health tools. Meanwhile, those who face structural inequalities are more likely to experience difficulty accessing high-quality reproductive health services and information.^37–39^ As a result, our findings may not represent the needs and concerns of underserved groups and non-female identifying persons facing barriers to miscarriage care in Canada. While this may limit generalizability, our findings are supported by work from Australia and the United States that endorse digital interventions for EPL.^27,29^

The qualitative interview and analysis process were also subject to the introduction of bias. Both interviewers were physicians with training in Obstetrics and Gynecology, which is necessarily imbued with perspectives around miscarriage care in Canada. Due to the virtual nature of the study collection, participants did not have the opportunity to review the interview transcripts or final study results. In order to minimize bias and support a wide range of perspectives, researchers were trained in qualitative methods, followed an open-ended interview guide, and incorporated divergent opinions into the reporting of the qualitative results.

## Conclusions

In this study, we explored the needs, desires, and preferences of women who recently experienced EPL. Participants were highly motivated to co-develop a digital intervention designed to fill gaps in miscarriage care in Canada. The digital companion would assist individuals through their miscarriage journey by providing evidence-based and locally relevant medical information as well as avenues to access psychosocial support. Informed by our findings, the next study phase will involve the creation and evaluation of a digital health tool designed to support people experiencing EPL.

## Declarations

**Ethics approval and consent to participate:** The study was approved by the Ottawa Health Science Network Research Ethics Board (OHSN-REB) (Protocol ID: 20210460-01H) on August 12, 2021. The OHSN-REB deemed our project “minimal risk” research, which it defines as research “in which the probability and magnitude of possible harms implied by participation in the research are no greater than those encountered by participants in those aspects of their everyday life that relate to the research.” The ethics board approved a Verbal Consent Script, which was read to each participant prior to the interview. The interviewer filled out an Informed Consent Form for each participant after the Verbal Consent Script was reviewed and the participant agreed to continue with the interview.

**Consent for publication:** Not applicable.

**Availability of data and materials:** The datasets generated during and/or analyzed during the current study are not publicly available due local ethics board regulations but are available from the corresponding author on reasonable request.
